# The Surgical Treatment of Abdominal Aneurysm

**Published:** 1904-06-25

**Authors:** 


### THE SURGICAL TREATMENT OF ABDOMINAL ANEURYSM.

Mr. Chas. Maunsell[1] describes the various surgical methods employed in the treatment of aneurysm of the abdominal aorta and its branches before the bifurcation, and critically considering these, comes to the conclusion that Corradi's modification of Moore's operation gives the best chance of cure.

Moore in 1864 introduced 26 yards of fine iron wire, by means of a cannula, into a thoracic aneurysm and by this means caused clotting of the blood in the sac, but the patient died of sepsis. Corradi passed 17 inches of annealed wire into a similar aneurysm, and then attached the anode terminal of a galvanic battery to this and allowed the current to flow for 25 minutes. The patient lived 13 weeks. Moore's operation had been performed 14 times in 1900 with 3 cures and 11 deaths. To this date 8 cases of abdominal aneurysm have been so treated with 3 cures.

Twenty-three aneurysms have been treated by Corradi's method with four cures, one considerably benefited and life prolonged, and ten in which death was hastened. In seven cases of abdominal aneurysm so treated there has been one cure, but the cases are too few for definite conclusions, and careful study of all cases, besides experimental data and scientific reasoning, leads steadily to the opinion that Corradi's modification is the better operation.

Hunner advocates using 10 to 20 feet of fine-drawn wire, 75 parts copper, 1,000 silver, passed through a cannula insulated by French lacquer. The anode must be applied to the wire and the cathode elsewhere, and not to the sac. The current should be from 20 to 60 milliampères, maintained for half-an-hour.

Mr. Maunsell relates a case under his own care; he treated a man aged 28, who had suffered from syphilis and developed an aneurysm of the aorta near the cœliac axis by the Moore-Corradi method. The abdomen was opened and the aneurysmal sac exposed; a purse-string suture enclosing an area the size of a shilling was passed, and a trocar with sealing-wax-covered cannula plunged into the aneurysm through the area of sac marked off by the suture. The trocar was withdrawn and 6 yards of sterilised No. 28 silver wire introduced, then the remaining portion of the wire on the reel was attached to the anode of a galvanic battery, the cathode communicated with gauze pads soaked in salt and water placed on the patient's sternum. The current was increased from 1 to 65 milliampères, and after half an hour again brought to zero. The wire was now cut short, the end pushed home, and the cannula withdrawn as the purse-string suture was tightened. During the passage of current the tumour became firmer, smaller, and the pulsation became less. For a fortnight the aneurysm seemed bigger, then it decreased rapidly and pulsation ceased. Forty-seven days after the operation the patient died of ulcer of the stomach perforating into the aneurysmal sac, which was small and nearly filled with firm laminated clot.

Of other methods of treating abdominal aneurysm tying the aorta has been done in 13 cases, all of which ended fatally. Temporary proximal ligature of the aorta has been suggested. Temporary distal compression has failed so far. Spontaneous cure is not unknown, but is very rare in large aneurysms. Starvation and rest, according to the methods of Bellingham and Tufnell, are rarely used except in connection with one of the more modern methods.

Large and increasing doses of iodide of potassium seem to do good. Calcium chloride is undoubtedly establishing itself in the favour of surgeons, but the gelatine treatment is hardly yet worth serious consideration. Macewen's needling method, and Velpeau's galvano-puncture are both uncertain, and the only safe method of using them is after laparotomy.

[1] Brit. Med. Jour., June 18, 1904.

